# Metformin and Risk of New-Onset Atrial Fibrillation in Type 2 Diabetes: A Systematic Review and Meta-Analysis

**DOI:** 10.3390/diagnostics15182288

**Published:** 2025-09-10

**Authors:** Roopeessh Vempati, Nanush Damarlapally, Poulami Roy, Maneeth Mylavarapu, Srivatsa Surya Vasudevan, Reshma Reguram, Tanisha Vora, Hritvik Jain, Raheel Ahmed, Geetha Krishnamoorthy

**Affiliations:** 1 Internal Medicine, Trinity Health Oakland Hospital, Pontiac, MI 48341, USA; reshmaraghuram49@gmail.com (R.R.); tanishavora98@gmail.com (T.V.); geetha.krishnamoorthy@trinity-health.org (G.K.); 2 Health Sciences, Houston Community College—Coleman Campus, Houston, TX 77030, USA; nanushraja@gmail.com; 3 Internal Medicine, North Bengal Medical College and Hospital, Siliguri 734012, India; poulami3613@gmail.com; 4 Baptist Health Walker Hospital, Jasper, AL 35510, USA; dr.maneeth.mylavarapu@gmail.com; 5 Louisiana State University Health Sciences Center, Shreveport, LA 71115, USA; srivatsa.suryavasudevan@gmail.com; 6 Internal Medicine, All India Institute of Medical Sciences, Jodhpur 342005, India; hritvikjain2001@gmail.com; 7 Cardiology, National Heart and Lung Institute, Imperial College London, London SW7 2AZ, UK; r.ahmed21@imperial.ac.uk

**Keywords:** atrial fibrillation, type 2 diabetes mellitus, metformin, cardiovascular outcomes, meta-analysis

## Abstract

**Background:** Atrial fibrillation (AF) is the most common sustained cardiac arrhythmia, increasingly prevalent worldwide. Type 2 diabetes mellitus (T2DM) is a major chronic disorder and a significant risk factor for AF, contributing to high morbidity and mortality. Metformin monotherapy can contribute to the reduced occurrence of adverse cardiovascular outcomes in patients with T2DM, but its effects on AF are understudied. This meta-analysis evaluates the association of metformin with the risk of incident AF among patients with T2DM on metformin. **Methods:** Databases, including PubMed, Google Scholar, and EMBASE, were screened through November 2024 for studies evaluating the association between metformin and new-onset AF in patients with T2DM. Comprehensive Meta-Analysis (CMA) version 4, by Biostat, Inc., utilizing a random effects model, was used to pool hazard ratios (HR) and 95% confidence intervals (CI). A meta-regression analysis was also performed to identify factors that may have influenced the results. A *p*-value < 0.05 was considered statistically significant. **Results:** A total of seven studies, comprising 4,017,929 patients with T2DM, having a mean age of 62.82 years and 52.5% males, were included. Metformin was associated with a statistically significantly lower risk of new-onset AF among patients with T2DM compared to other hypoglycemic agents (aHR: 0.85; 95% CI 0.76–0.94; *p* = 0.002). Meta-regression analysis identified age as a significant moderator of the treatment effect (β = −3.15, *p* = 0.001). **Conclusions:** Metformin is associated with a lower risk of new-onset AF among patients with T2DM compared to other hypoglycemic agents. Furthermore, age-related attenuation of this association was observed, with older patients with T2DM showing a weaker association.

## 1. Introduction

The estimated global prevalence of atrial fibrillation (AF) reached 50 million in 2020. Furthermore, many patients remain undiagnosed. The increasing burden of AF worldwide is multifactorial, attributable to factors such as an aging population, the rising incidence of obesity, improved detection methods, and higher survival rates among patients with AF and other cardiovascular diseases [1]. In the United States, the AF burden was recorded at 5.2 million in 2010, with projections estimating that it will rise to 12.1 million by 2030 [2]. Type 2 diabetes mellitus (T2DM) affects 38.4 million people in the United States [3]. T2DM stands as one of the most common chronic disorders globally and is associated with increased mortality and disability. It is linked to numerous cardiovascular adverse events, including atrial and ventricular arrhythmias, which can lead to poor outcomes [4].

Inflammation has been identified as the underlying pathogenic mechanism of AF in patients with T2DM [5,6]. Research shows that insulin resistance and diabetes can cause structural, electrical, electromechanical, and autonomic remodeling in the atria, leading to arrhythmogenic substrates for AF [5,7]. AF has been found to correlate with elevated oxidative stress and induce structural remodeling in atrial myocytes, resulting in the degradation of myofibrils and glycogen accumulation [8]. Metformin has demonstrated anti-inflammatory properties and reduces inflammation and oxidative stress, independent of its anti-hyperglycemic effects [9,10,11,12,13]. Studies have long reported that metformin reduces cardiovascular deaths in patients with T2DM [14,15,16,17,18].

However, the risk of incident AF among patients with T2DM on metformin remains understudied. To address this knowledge gap, a comprehensive systematic review and meta-analysis were performed to evaluate the association between metformin use and the risk of incident AF among patients with T2DM. This study aims to provide clinicians with evidence-based guidance for optimizing treatment strategies and improving cardiovascular outcomes in this vulnerable population. To our knowledge, this is the first meta-analysis to evaluate this association.

## 2. Methods

We conducted our meta-analysis per the Preferred Reporting Items for Systematic Reviews and Meta-Analyses (PRISMA) guidelines [19]. The study protocol was registered with the OSF (Open Science Framework) Registries ID: osf.io/43sr9.

### 2.1. Data Sources and Search Strategy

Studies examining the association between metformin and new-onset AF in T2DM were thoroughly searched from databases such as PubMed, Google Scholar, and Embase until November 2024. We used MeSH terms and keywords, including “atrial fibrillation,” “metformin,” and “type 2 diabetes mellitus,” which were utilized during our search process. A detailed search strategy was reported in Supplementary Table S1.

### 2.2. Inclusion and Exclusion Criteria

**Figure 1:**
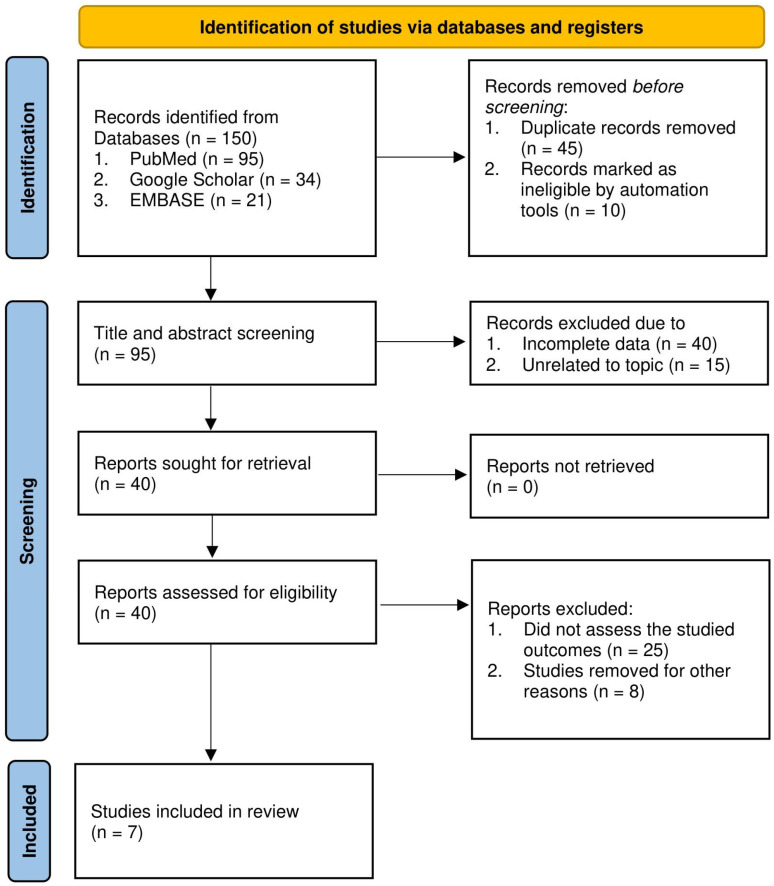
Study selection process of included studies.

Articles retrieved from the databases using the search strategy were imported into Rayyan. Duplicates were removed, and title and abstract screening were performed by two independent reviewers, RV and ND, with discrepancies resolved by a third reviewer, SV. During the selection process, we aimed to include prospective and retrospective cohort studies, case–control studies, and other observational designs that reported adjusted hazard ratios (aHRs), 95% confidence intervals (CIs), and/or their respective *p*-values. We excluded non-English studies, reviews, studies lacking effect size measures, animal studies, case reports, and editorials from the analysis. Figure 1 outlines the detailed study selection process. Detailed inclusion and exclusion criteria were reported in Supplementary Table S2.

### 2.3. Data Extraction and Quality Assessment

Two reviewers (RV and ND) independently extracted data from the finalized studies using a Microsoft Excel sheet. Full texts were screened for factors such as demographics and clinical characteristics, and HRs regarding the association of risk of AF among patients with T2DM were extracted. Comparator groups varied across studies (e.g., non-metformin users, no therapy, or active comparators such as sulfonylureas, DPP-4 inhibitors, GLP-1 receptor agonists, and thiazolidinediones); details are provided in Supplementary Table S3. For each study, AF definitions and diagnostic methods (e.g., ICD codes, hospitalization records, or device monitoring) are detailed in Supplementary Table S4. The risk of bias assessment was conducted using the adaptations of Newcastle-Ottawa (NOS) and visualized using the Robvis software, with the domains, potential confounding (D1), exposure measurement (D2), participant selection (D3), post-exposure interventions (D4), missing data (D5), outcome measurement (D6), and selective reporting (D7) [20].

### 2.4. Statistical Analysis

All the statistical analyses were performed using the Comprehensive Meta-Analysis (CMA) (Biostat, Inc., Englewood, NJ, USA) [21] with a random effects model for pooling aHRs and 95% CI. A leave-one-out sensitivity analysis was performed to assess the robustness of the results, and I^2^ statistics were used to study the heterogeneity in the effect estimate. Heterogeneity was categorized as follows: I^2^ of 25–50% was deemed mild, 50–75% moderate, and >75% severe heterogeneity. Forest plots were created depicting the effect measures of each study, along with pooled HRs and leave-one-out sensitivity analysis. A meta-regression analysis was performed to study any confounding factors. A *p*-value < 0.05 was considered to be statistically significant. Funnel plots were utilized to evaluate for publication bias, recognizing that asymmetry may suggest publication bias.

### 2.5. Ethical Considerations

This study was based on a systematic review of previously published studies, so no ethical approval was required. All included studies were assumed to have obtained appropriate ethical approval.

## 3. Results

### 3.1. Baseline Characteristics

Seven studies [4,6,22,23,24,25,26] were included in our analysis. The majority of them were retrospective cohorts (*n* = 5), followed by cross-sectional (*n* = 1) and case–control studies (*n* = 1). The 4,017,929 patients with T2DM had a mean age of 62.82 (five studies), and the majority (six out of seven studies) were males. Table 1 outlines the baseline characteristics of the included studies.

### 3.2. Metformin and Risk of Atrial Fibrillation

**Figure 2:**
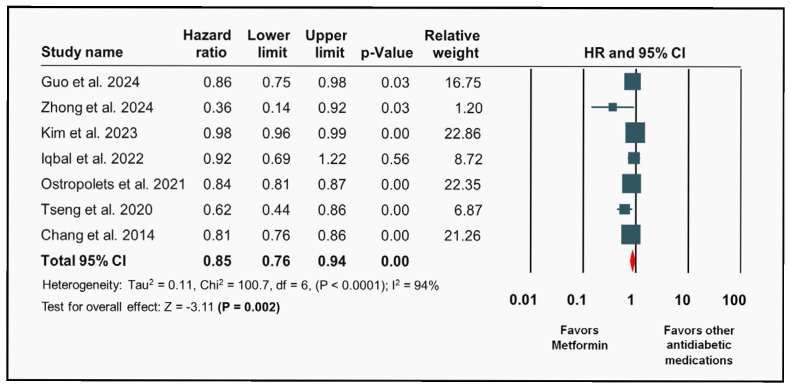
Association of metformin with new-onset atrial fibrillation. Forest plot illustrating the association of metformin with new-onset atrial fibrillation outcomes [4,6,22,23,24,25,26].

**Figure 3:**
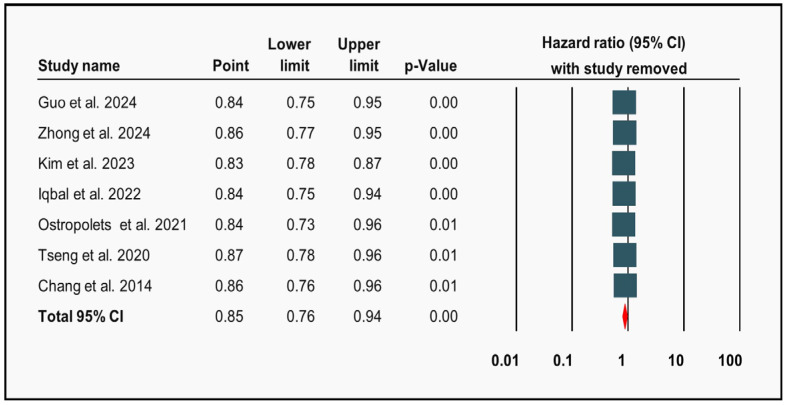
Leave-one-out sensitivity analysis. Forest plot illustrating that no single study is exerting a disproportionate influence on the overall effect estimate [4,6,22,23,24,25,26].

Metformin was associated with a statistically significant lower risk of new-onset AF in patients with T2DM compared to other hypoglycemic medications (aHR: 0.85; 95% CI 0.76–0.94; *p* = 0.002) (Figure 2). However, the analysis also revealed high heterogeneity (I^2^: 94%). Notably, comparator definitions varied across studies (Supplementary Table S3), which may have contributed to the observed heterogeneity. A leave-one-out sensitivity analysis and upon removal of individual studies, the HR and CI remained within a narrow range, indicating that no single study exerted undue influence on the overall effect estimate, and consistent results across all iterations (Figure 3).

### 3.3. Meta-Regression and Publication Bias

**Figure 4:**
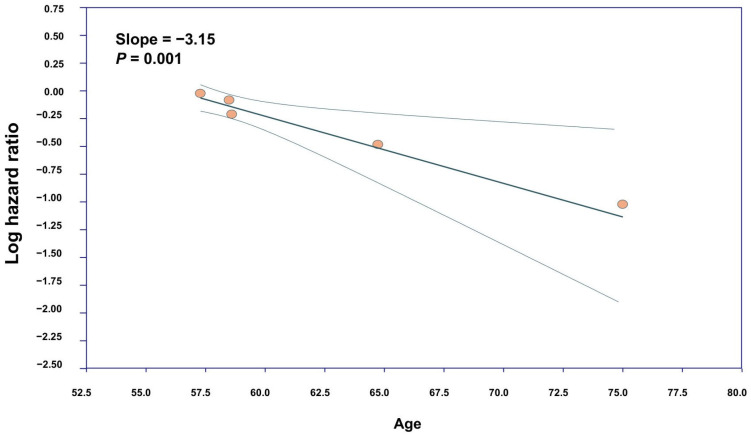
Meta-regression analysis. Scatter plot illustrating meta-regression analysis of age as a factor.

**Figure 5:**
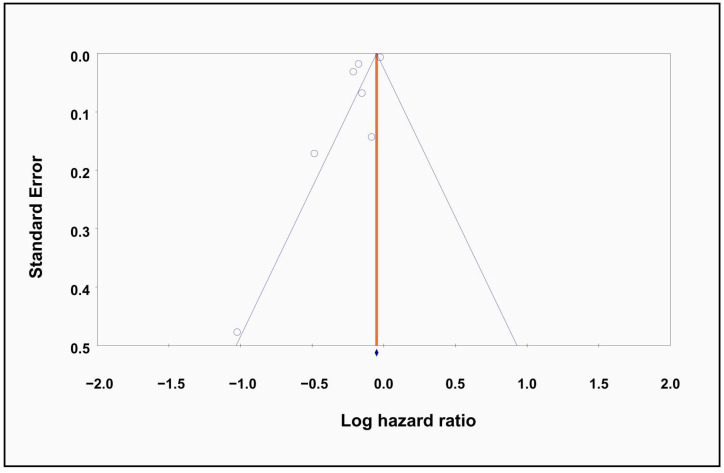
Publication bias assessment. Funnel plot for publication bias assessment.

Meta-regression analysis revealed age to be a significant moderator of treatment effect, which implied that the association of metformin with new-onset AF decreases with increasing age (β = −3.15, *p* = 0.001), indicating a significantly weaker association in older compared to younger patients with T2DM (Figure 4). There was no significant association between males (*p* = 0.30) and other prognostic factors, such as hypertension (HTN) (*p* = 0.72) or stroke (*p* = 0.45), and our primary outcomes. Visualization of the funnel plot demonstrated minor asymmetry. Given the small number of included studies (<10), funnel plot interpretation remains limited, as standard asymmetry tests are underpowered in this context (Figure 5).

### 3.4. Risk of Bias Assessment

**Figure 6:**
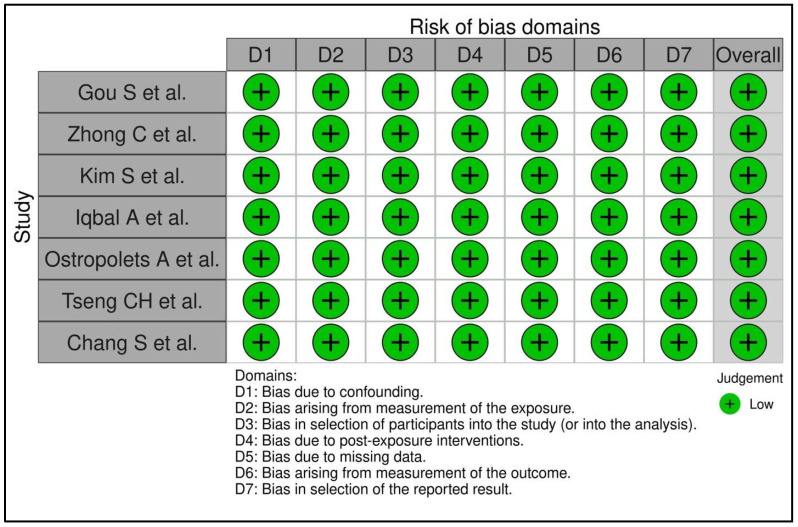
Risk of bias assessment of included studies. Traffic light plot for risk of bias assessment [4,6,22,23,24,25,26].

The risk of bias assessment revealed that all included studies were at low risk of bias. Each study adequately addressed potential confounding (D1), exposure measurement (D2), participant selection (D3), post-exposure interventions (D4), missing data (D5), outcome measurement (D6), and selective reporting (D7). Figure 6 depicts the RoB assessment for the included studies.

## 4. Discussion

Our meta-analysis, encompassing a large sample of 4,017,929 patients with T2DM, demonstrates that metformin is associated with a significantly lower risk of new-onset AF compared to other hypoglycemic medications. These findings are particularly noteworthy given the global burden of both T2DM and AF, which collectively contribute to substantial morbidity and mortality [27,28]. This is the first meta-analysis examining the association between metformin and the risk of new-onset AF, and the current literature on this association is limited. However, Liou et al. [29] reported that, among antihyperglycemic agents, biguanides are linked to the second-lowest risk of new-onset AF in patients with T2DM, following thiazolidinediones.

### 4.1. Interpretation of Individual Studies

Almost all included studies [4,6,22,23,24,25,26] support the hypothesis that metformin reduces the risk of new-onset AF. Guo et al. [22] found that metformin use, compared to other hypoglycemic medications, was associated with a lower risk of AF, particularly in non-insulin users. Similarly, Kim et al. [23] showed that metformin monotherapy and combination therapy significantly reduced AF incidence compared to no medication, with metformin plus thiazolidinediones showing an even stronger effect across various subgroups, including age, sex, and diabetes severity. Tseng et al. [25] provided further evidence from a large Taiwanese cohort, showing a dose–response relationship between cumulative metformin use and reduced hospitalization rates for AF. Chang et al. [6] complemented these findings by reporting that metformin independently decreased AF risk and provided mechanistic insights, demonstrating that metformin attenuates atrial cell oxidative stress and myolysis. Ostropolets et al. [4] observed reduced atrial arrhythmias, including AF, with metformin monotherapy compared to sulfonylureas and DPP4 inhibitors, but cautioned against metformin–sulfonylurea combination therapy. Zhong et al. [26] found that metformin was associated with a lower risk of new-onset AF in patients without prior arrhythmias, but the effect was limited to preventing new cases rather than reducing the burden of AF in those already affected. In contrast, Iqbal et al. [24] reported no significant association between metformin and reduced AF risk, possibly due to other risk factors like age and heart failure, as well as study limitations. While most studies [4,6,22,23,24,25,26] suggest that metformin is associated with a lower risk of new-onset AF, with its anti-inflammatory, metabolic, and cardiovascular benefits, findings from Iqbal et al. [24] highlight the complexity of AF pathogenesis and the potential influence of unmeasured confounders and competing risks.

### 4.2. Pathophysiology of Development of Atrial Fibrillation in Type 2 Diabetes Mellitus

T2DM is recognized as an independent risk factor for AF, contributing to its development through complex pathophysiological processes. T2DM promotes AF through a combination of structural, electrical, electromechanical, and autonomic remodeling. Chronic hyperglycemia and glycemic variability induce oxidative stress and inflammation, which are central to these changes. Oxidative stress in T2DM drives atrial fibrosis via mitochondrial dysfunction and activation of pro-fibrotic TGF-β pathways [30,31]. Advanced glycation end products (AGEs) and their receptors upregulate connective tissue growth factor, exacerbating atrial fibrosis and dilatation, which serve as substrates for AF initiation and maintenance [32,33]. Electrical remodeling involves prolongation of atrial effective refractory periods, decreased sodium channel (SCN5A) expression, and connexin dysregulation, creating a pro-arrhythmic atrial substrate [34,35,36]. Electromechanical remodeling impairs excitation–contraction coupling, leading to delayed atrial conduction and impaired atrial emptying [37,38]. Autonomic neuropathy, characterized by parasympathetic denervation and heightened sympathetic activity, destabilizes atrial rhythm further, increasing AF susceptibility [39,40]. Together, these processes highlight the complex interplay between T2DM and AF pathogenesis [7,34]. Emerging evidence indicates that arterial stiffness is an early and modifiable marker of cardiovascular risk, particularly in individuals with metabolic disturbances. Emerging evidence also links vascular remodeling and arterial stiffness to arrhythmia susceptibility, underscoring the multifactorial nature of AF in T2DM [41].

### 4.3. Association of Metformin Against New-Onset Atrial Fibrillation in Type 2 Diabetes Mellitus

Metformin directly counteracts several of the DM-related pathways described above, supporting its observed association with reduced AF risk. Metformin increases adiponectin levels, which improves insulin sensitivity by reducing hepatic glucose production and promoting fatty acid oxidation [40,42]. Additionally, metformin’s role in anti-inflammatory pathways is supported by the increased macrophage activation marker CD68 levels observed in these patients [40].

**Figure 7:**
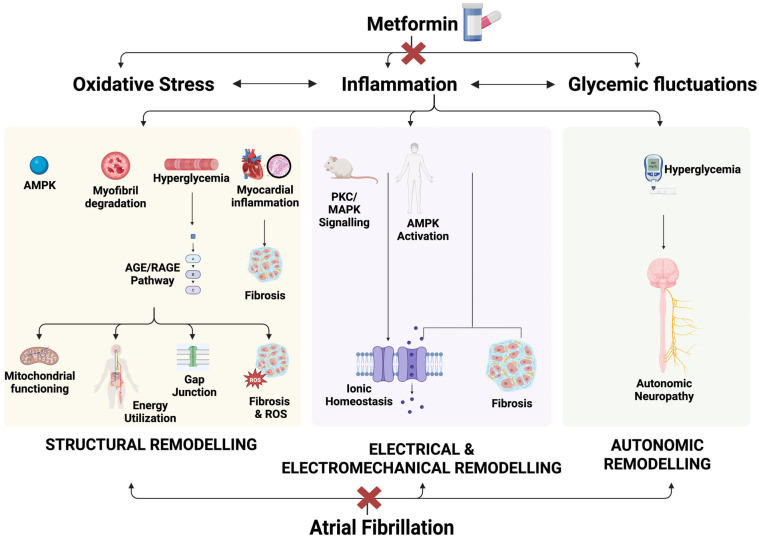
Metformin’s actions on atrial fibrillation. AGE/RAGE, Advanced Glycation End products/Receptor of Advanced Glycation End products; AMPK, AMP-activated Protein Kinase; ROS, Reactive Oxygen Species; PKC, Protein Kinase C; MAPK, Mitogen-Activated Protein Kinase.

Metformin reduces lipid accumulation in cardiac tissues, as shown in diabetic patients who received non-diabetic heart transplants [43]. Animal models further corroborate this reduction in lipid deposition. In a swine model of ischemia, metformin enhanced AMP-activated protein kinase (AMPK) activation and maintained myocardial ATP concentrations, lowering mortality from ventricular fibrillation [44]. Similarly, a canine model of acute AF demonstrated that metformin-mediated AMPK activation decreased fatty acid deposition in the left atrial appendage by upregulating metabolic proteins such as PPARα, CPT-1, and VLCAD [45]. Metformin also has anti-fibrotic effects, which are crucial for preventing AF. In obese animal models, metformin reduced fibrosis, while in T2DM rats, it inhibited the PKC/ERK signaling pathway and preserved the expression of small-conductance calcium-activated potassium (SK) channels, whose downregulation is associated with AF [46,47]. Figure 7 illustrates key pathways in the potential mechanism of action of AF.

### 4.4. Sensitivity and Meta-Regression Analyses

Despite considerable heterogeneity, our findings were robustly affirmed through a leave-one-out sensitivity analysis that demonstrated a consistent association of metformin with a reduced occurrence of AF across all iterations. Furthermore, the meta-regression analysis identified age as a significant moderator of treatment effect, revealing a diminishing association of metformin and risk of new-onset AF with increasing age. This observation aligns with prior evidence suggesting that aging-associated atrial remodeling and comorbidities may attenuate the efficacy of pharmacological interventions. It also raises important questions about whether older adults require tailored therapeutic strategies to optimize outcomes.

In the included studies, hypertension and stroke were among the common comorbidities (Table 1), and both have relevant associations with AF. Atrial remodeling caused by HTN is characterized by structural and electrical changes [1]. Stroke, though it is traditionally viewed as a consequence of AF, can also indicate underlying pathology of the atria and can trigger AF through post-stroke autonomic dysregulation, particularly with insular involvement [1,39]. In our meta-regression analysis, neither HTN (*p* = 0.72) nor stroke (*p* = 0.45) significantly reduced the association between metformin and new-onset AF, suggesting that the observed association is independent of these comorbidities. However, residual confounding cannot be excluded, given the heterogeneity in the adjustment across the studies.

### 4.5. Strengths and Limitations

The strengths of this meta-analysis include its large sample size, rigorous methodology, and comprehensive statistical analyses that address confounding factors and heterogeneity. Additionally, we employed a random effects model, conducted a leave-one-out sensitivity analysis, and performed a meta-regression analysis to support our findings.

However, several limitations need to be considered. The included studies were mainly observational, which may introduce residual confounding despite adjustments for baseline characteristics. Comparator definitions differed across studies (Supplementary Table S3), precluding a uniform head-to-head meta-analysis and likely contributing to heterogeneity. Therefore, we were unable to establish a matched control group or assess relative risk across standardized populations. Heterogeneity (I^2^ = 94%) may reflect differences in study design, populations, AF ascertainment, and healthcare systems. The heterogeneity in AF definitions and variations in follow-up durations may affect generalizability. In addition, inconsistent reporting of diabetes duration, glycemic control, and medication adherence, along with limited data on concurrent AF-modifying therapies (e.g., beta-blockers, calcium channel blockers, sodium–glucose transportase-2 inhibitors (SGLT2i), glucagon-like receptor agonists (GLP-1 RA), and anti-arrhythmics), raises concern for residual confounding and limits causal inference (Supplementary Table S5). Furthermore, information on AF detection methods, such as Holter monitoring or continuous rhythm surveillance, was lacking in the majority of studies, and most of the studies, owing to a database origin, relied on ICD codes, which may under-capture AF, as noted in Supplementary Table S4. Crucially, echocardiographic parameters, such as left atrial diameter, left ventricular ejection fraction, valvular abnormalities, and pulmonary artery pressure, were not consistently available, limiting our ability to account for structural heart disease as a contributor to AF. Similarly, key clinical variables such as thyroid function tests and infectious or inflammatory markers were absent across studies, despite their established role in AF pathophysiology. These gaps highlight the residual confounding and the need for prospective studies with detailed clinical and imaging data to better delineate the relationship between metformin and new-onset AF. While most of the studies we included demonstrated a positive relationship between metformin and a reduced risk of AF, we decided to include all eligible studies identified in our search, regardless of their findings. This decision may still indicate a publication bias, as studies with negative results are often underreported. Our meta-analysis enhances the understanding of this topic by integrating evidence from various populations and study designs, ultimately providing a more reliable overall estimate. In addition, due to the limited number of studies and variability in reporting, subgroup analyses by study design, region, or AF detection methods could not be robustly performed. Furthermore, this meta-analysis could not identify a definitive cut-off age at which metformin’s association with reduced occurrence of new-onset AF declines, likely due to individual studies not addressing this age-related decline and the limited number of studies available for subgroup analysis on this specific question.

### 4.6. Clinical Implications

The clinical implications of the current findings are extensive. For clinicians managing patients with T2DM, metformin’s association with new-onset AF provides an additional justification for its preferred use, especially in younger and middle-aged adults. It is important to acknowledge that metformin is not an anti-arrhythmic drug, and the anti-arrhythmic effect of metformin is due to its anti-inflammatory, metabolic, and anti-fibrotic effects [42,43,44,45,46]. While metformin’s anti-inflammatory, anti-fibrotic, and metabolic effects provide biologic plausibility for AF risk reduction, the modest pooled effect size (HR 0.85) suggests that residual confounding may also contribute. Differences in diabetes duration, HbA1c, and cardiovascular medication use, variably adjusted across studies [4,6,22,23,24,25,26] (Supplementary Table S5), may partially account for the observed association, highlighting the need for cautious interpretation. Metformin remains a foundational therapy in T2DM, particularly due to its safety and metabolic profile. However, recent guidelines no longer prescribe it as mandatory first-line therapy for all patients. In individuals with cardiovascular or renal comorbidities, SGLT-2 inhibitors or GLP-1 receptor agonists may be prioritized in conjunction with or even ahead of metformin [48]. However, metformin is weight-neutral or even associated with modest weight loss, contrasting sharply with the weight gain commonly observed with thiazolidinediones [49].

Furthermore, metformin carries a lower risk of fluid retention and edema, a significant concern with thiazolidinediones, particularly in patients with heart failure [50,51]. While both drug classes can impact cardiovascular health, some studies suggest that metformin may have a potentially beneficial effect on cardiovascular outcomes. In contrast, thiazolidinediones have been associated with an increased risk of certain cardiovascular events, especially in susceptible individuals [52]. Prior investigations have highlighted metformin as a compelling candidate among potential drugs repurposed for AF treatment [53]. However, the age-related reduction in the association of metformin and risk of new-onset AF highlights the necessity for personalized treatment plans that consider patient-specific factors such as age, duration of diabetes, and existing cardiovascular risk profiles. In older patients, additional strategies aimed at other modifiable risk factors for AF, such as blood pressure control and lipid management, may be required to further optimize the risk.

### 4.7. Future Directions

The results of this meta-analysis pave the way for further research to enhance the current understanding of the relationship between metformin and AF. Future studies should aim to categorize patients by age, diabetes duration, and other key variables to identify subgroups in whom metformin is more strongly associated with lower AF risk. Additionally, prospective studies and randomized controlled trials with standardized definitions of AF and thorough long-term follow-up are needed to validate our findings and address the limitations inherent in observational data. Incorporating echocardiographic parameters, biomarkers, and comprehensive cardiovascular risk profiles will be essential to disentangle the independent effects of metformin from underlying AF pathophysiology.

## 5. Conclusions

Our meta-analysis revealed that, compared to other hypoglycemic agents, metformin use is associated with a significantly lower risk of new-onset AF in patients with T2DM. Furthermore, we also observed an age-related attenuation of this association. The anti-inflammatory, anti-fibrotic, and metabolic effects of metformin likely contribute to decreasing the likelihood of new-onset AF as they combat the pathophysiology of AF in T2DM. In addition to patient-specific factors, clinicians should consider the potential cardiovascular benefits of metformin to optimize diabetes management and prevent AF. However, further randomized controlled trials are necessary to confirm these findings and explore age-related treatment disparities due to the observational nature of the included studies, as well as variations in AF definitions and follow-up durations. The absence of individual-level echocardiographic and hormonal data, as well as unmeasured confounders, highlights the need for prospective, well-controlled studies with detailed cardiac and metabolic profiling to confirm metformin’s impact on AF risk.

## Figures and Tables

**Table 1:** Baseline characteristics of the study population in the included studies.

Author	Study Design	Country	Type	Size	Age, in Years (Mean ± SD)	Female *n* (%)	Duration, in Years	Co-Morbidities					
								Alcohol *n* (%)	Smoking *n* (%)	HTN *n* (%)	HF *n* (%)	Stroke *n* (%)	CKD *n* (%)
Gou et al., 2024 [22]	CS	UK	M	10,011	-	-	Until 2023	-	-	-	-	-	-
Zhong et al., 2024 [26]	CC	China	S	227	74.87 ± 9.2	93 (40.97%)	4	24 (10.5)	42 (18.5)	169 (74)	33 (14.5)	35 (15.4)	53 (23.3)
Kim et al., 2023 [23]	RC	South Korea	M	2,515,468	57.28 ± 11.72	1,000,904 (39.79%)	9	252,222 (10)	658,980 (26.2)	1,414,087 (56.2)	34,009 (1.35)	111,906 (4.45)	283,816 (11.3)
Iqbal et al., 2022 [24]	RC	USA	S	5664	61.39 ± 3.39	3311 (58.45%)	10	662 (11.7)	649 (11.5)	3530 (62.3%)	107 (1.89)	197 (3.5)	19 (0.34)
Ostropolets et al., 2021 [4]	RC	USA	M	645,785	NR	325,504 (48.9%)	2.7	NR	NR	133,221 (70)	8875 (4.67)	NR	18,542 (9.75)
Tseng 2020 [25]	RC	Taiwan	M	195,064	64.73 ± 12.21	91,702 (47.0%)	5	12,849 (6.6)	6946 (3.5)	163,385 (83.8)	NR	104,965 (53.8)	NR
Chang et al., 2014 [6]	RC	Taiwan	M	645,710	58.6 ± 17.1	93,014 (50.4%)	5.4	NR	NR	82,081 (58)	12,563 (8.9)	14,557 (10.3)	25,237 (17.9)

Legend: CS, Cross-Sectional; CC, Case–Control; CKD: Chronic Kidney Disease; HTN, Hypertension; HF, Heart Failure; RC, Retrospective Cohort; SD, Standard Deviation; USA, United States of America; UK: United Kingdom; NR, Not reported.

## Data Availability

The raw data supporting the conclusions of this article will be made available by the authors on request.

## Supplementary Materials

The following supporting information can be downloaded at: https://www.mdpi.com/article/10.3390/diagnostics15182288/s1, Supplementary Table S1: Search Strategy; Supplementary Table S2: Inclusion & Exclusion Criteria; Supplementary Table S3: Comparator group across the included studies; Supplementary Table S4: Atrial fibrillation ascertainment method and definition across the included studies; Supplementary Table S5: Confounders adjusted in the included studies.

## Abbreviations

The following abbreviations are used in this manuscript:

AF	Atrial Fibrillation
T2DM	Type 2 Diabetes Mellitus
CMA	Comprehensive Meta-Analysis
HR	Hazard Ratio
CI	Confidence Interval
PRISMA	Preferred Reporting Items for Systematic Reviews and Meta-Analyses
OSF	Open Science Framework
MeSH	Medical Subject Headings
CS	Cross-Sectional (study design)
CC	Case–Control (study design)
CKD	Chronic Kidney Disease
HTN	Hypertension
HF	Heart Failure
RC	Retrospective Cohort (study design)
SD	Standard Deviation
USA	United States of America
ROS	Reactive Oxygen Species
TGF-β	Transforming Growth Factor-beta
AGEs	Advanced Glycation End products
SCN5A	Sodium Channel (gene/protein)
AMPK	AMP-activated Protein Kinase
PPARα	Peroxisome Proliferator-Activated Receptor Alpha
CPT-1	Carnitine Palmitoyltransferase 1
VLCAD	Very-long-chain acyl-CoA dehydrogenase
PKC	Protein Kinase C
ERK	Extracellular signal-regulated kinase
SK	Small-conductance Calcium-activated Potassium (channel)
MAPK	Mitogen-Activated Protein Kinase
