# Supplementary material for: Metformin and Risk of New-Onset Atrial Fibrillation in Type 2 Diabetes: A Systematic Review and Meta-Analysis
Source: Diagnostics (Basel). 2025 Sep 10;15(18):2288. doi: 10.3390/diagnostics15182288 (PMC12468145; doi:10.3390/diagnostics15182288)
Supplement: Supplementary file 1 [file diagnostics-15-02288-s001.zip › diagnostics-3815776-supplementary.pdf]

**Supplementary File S1. Search Strategy**

| Database | Search Terms |
|---|---|
| PubMed (NLM) | ("atrial fibrillation"[MeSH Terms] OR "AF") AND ("metformin"[MeSH Terms]) AND ("type 2 diabetes mellitus"[MeSH Terms] OR "T2DM" OR "diabetes mellitus") |
| Embase (Elsevier) | ('atrial fibrillation'/exp OR 'atrial fibrillation') AND ('metformin'/exp OR 'metformin') AND ('type 2 diabetes mellitus'/exp OR 'type 2 diabetes mellitus' OR 'T2DM' OR 'diabetes mellitus') |
| Google Scholar (Google) | "atrial fibrillation" AND "metformin" AND ("type 2 diabetes mellitus" OR "T2DM") |

**Supplementary File S2. Inclusion & Exclusion Criteria**

| Criteria | Inclusion | Exclusion |
|---|---|---|
| Study Design | Observational studies (cohort, case-control, cross-sectional) | Reviews, meta-analyses, editorials, letters, conference abstracts (except for searching conference abstracts as grey literature), animal studies, in vitro studies |
| Population | Patients with type 2 diabetes mellitus | Studies not including patients with T2DM |
| Intervention/Exposure | Metformin use | Studies not investigating metformin use |
| Comparison | Other hypoglycemic agents, placebo, no treatment | N/A |
| Outcome | New-onset atrial fibrillation (defined as the first documented episode of AF) | Studies focusing on AF recurrence or burden, rather than new-onset AF |
| Language | English | Studies not published in English |
| Publication Status | Published studies, Preprints, Conference Abstracts, Grey Literature | |
| Data Reporting | Reporting of hazard ratios (HRs) or odds ratios (ORs) with 95% confidence intervals (CIs) or p-values for the association of interest | Studies not reporting relevant effect estimates |

**Supplementary File S3. Comparator group across the included studies**

| Author | Comparision |
|---|---|
| Gou S et al 2024 [22] | other anti-diabetics |
| Zhong C et al 2024 [26] | Non-users of metformin |
| Kim S et al 2023 [23] | Metformin combination/ other drugs |
| Iqbal A et al 2022 [24] | other than metformin |
| Ostropolets A et al 2021 [4] | DPP4i, GLP-1 RA, SUs, TZDs, and never metformin users |
| Tseng CH 2020 [25] | Non metformin |
| Chang S 2014 [26] | Non metformin |
| DPP4i: dipeptidyl peptidase-4 inhibitors, GLP-1 RA: glucagon-like peptide-1 receptor agonists, SUs: sulfonylureas, TZDs: thiazolidinediones | |

**Supplementary File S4. Atrial fibrillation ascertainment method and definition across the included studies**

| Author (Year) | AF Ascertainment Method | Notes on Definition |
|---|---|---|
| Guo S et al. 2024 [22] | ICD-10 codes from linked hospital/primary care records | Incident AF is defined by diagnostic codes |
| Zhong C et al. 2024 [26] | Continuous device-detected AF (pacemaker diagnostics) | Captured subclinical and paroxysmal AF |
| Kim S et al. 2023 [23] | ICD-10 codes from insurance claims | New AF defined by ≥1 inpatient or ≥2 outpatient diagnoses |
| Iqbal A et al. 2022 [24] | ICD-9 and 10 codes in EHR/claims | Incident AF adjudicated with competing risk models |
| Ostropolets A et al. 2021 [4] | ICD-9/10 diagnostic codes | Based on validated code lists |
| Tseng C.H. 2020 [25] | Hospitalization for AF (ICD-9-CM 427.31, ≥2 claims) | AF diagnosis required ≥2 encounters or admission |
| Chang S. 2014 [6] | ICD-9-CM 427.31 recorded on ≥2 occasions | Incident AF required repeat coding to reduce misclassification |

**Supplementary File S5. Confounders adjusted in the included studies**

| Author (Year) | Key Confounders Adjusted For |
|---|---|
| Guo S et al. 2024 [22] | Age, sex, race, SBP, DBP, smoking, BMI, MI, HF, anti-HTN meds, HbA1c, T2DM duration |
| Zhong C et al. 2024 [26] | Model 1: Metformin use, age, sex; Model 2: + BMI, alcohol, smoking; Model 3: + LAD, Cr, complications (HTN, HF, stroke, CKD, CVD, MI), meds (insulin, SU, TZD, DPP4i, GLP1-RA, SGLT2i, statins, ACEi/ARB, etc.) |
| Kim S et al. 2023 [23] | Model 1: Age, sex, BMI; Model 2: + income, living area, smoking, alcohol, exercise; Model 3: + glucose, T2DM duration; Model 4: + comorbidities (HTN, DL, CKD, HF, MI, stroke, PAD) |
| Iqbal A et al. 2022 [24] | Age, sex, race, smoking, BMI, LDL, HDL, TG, HbA1c, anti-hyperglycemic class (metformin, SU, TZD, AGI, DPP4i, GLP1-RA, SGLT2i, meglitinides, insulin) |
| Ostropolets A et al. 2021 [4] | Baseline demographics (age strata, sex), comorbidities (HTN, CAD, HF, valvular disease, hypothyroidism, hyperparathyroidism, CKD, COPD, asthma), and meds (beta-blockers, CCB, ACEi, anticoagulants, statins). Balance achieved after PS adjustment (standardized differences <0.01). |
| Tseng C.H. 2020 [25] | Demographics (age, sex, occupation, region); major comorbidities (HTN, DL, obesity); diabetes complications (nephropathy, eye disease, stroke subtypes, IHD, PAD); antidiabetic drugs (insulin, SU, meglitinides, AGI, rosiglitazone, pioglitazone); AF risk factors (COPD, smoking, alcohol-related dx, cancer, HF, gout, hyperthyroidism, sleep apnea, valvular disease); common meds (ACEi/ARB, CCB, beta-blockers, statins, fibrates, aspirin) |
| Chang S. 2014 [6] | Age, sex, HTN, CHF, CKD, asthma, MI, ischemic stroke, PAD, meds (anti-HTN, statins) |

SBP: systolic blood pressure; DBP: diastolic blood pressure; BMI: body mass index; MI: myocardial infarction; HF: heart failure; CHF: congestive heart failure; anti-HTN meds: antihypertensive medications; HbA1c: glycated hemoglobin; T2DM: type 2 diabetes mellitus; LAD: left atrial diameter; Cr: creatinine; CKD: chronic kidney disease; CVD: cardiovascular disease; SU: sulfonylureas; TZD: thiazolidinediones; DPP4i: dipeptidyl peptidase-4 inhibitors; GLP1-RA: glucagon-like peptide-1 receptor agonists; SGLT2i: sodium-glucose co-transporter 2 inhibitors; ACEi/ARB: angiotensin-converting enzyme inhibitor/angiotensin receptor blocker; DL: dyslipidemia; PAD: peripheral arterial disease; CAD: coronary artery disease; IHD: ischemic heart disease; COPD: chronic obstructive pulmonary disease; CCB: calcium channel blocker; LDL: low-density lipoprotein cholesterol; HDL: high-density lipoprotein cholesterol; TG: triglycerides; AGI: α-glucosidase inhibitors; PS: propensity score.
